# Supplementary material for: Dynamic regulation of mitotic ubiquitin ligase APC/C by coordinated Plx1 kinase and PP2A phosphatase action on a flexible Apc1 loop
Source: EMBO J. 2021 Jul 22;40(18):e107516. doi: 10.15252/embj.2020107516 (PMC8441438; doi:10.15252/embj.2020107516)
Supplement: Supplementary file 2 — Appendix [file EMBJ-40-e107516-s001.pdf]

Appendix

Table of Contents 1

Appendix Figure S1 2

Appendix Figure S2 3

Appendix Figure S3 4

Appendix Figure S4 5

Appendix Figure S5 6

Appendix Figure S6 7

Appendix Figure S7 8

Appendix Figure S8 9

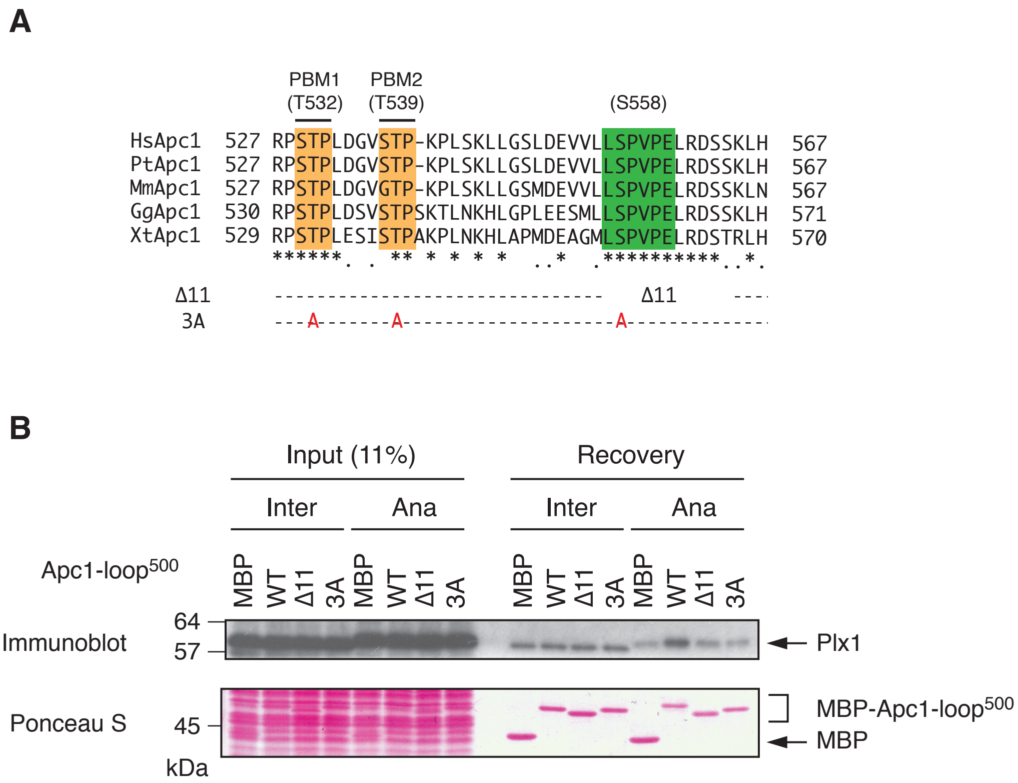

Appendix Fig S1. Plx1 binds to Apc1-loop500 in anaphase, but not in interphase.
A. Multiple alignment of sequences of Apc1-loop500 containing PBMs (Orange) and B56-binding site (green) are shown as Fig 1A. Eleven-residue [LSPVPELRDST] deletion including the B56-binding site (Δ11) and alanine substitution mutations to three CDK phosphorylation sites (3A) are indicated.
B. Apc1-loop500 binding assay. Apc1-loop500 WT or its derivatives (Δ11 or 3A) were incubated with interphase extracts or anaphase extracts supplemented with non-degradable cyclin B at 23˚C for 1 h. The bound proteins were recovered by amylose beads, separated by SDS–PAGE and detected by immunoblotting with Plx1 antibody or Ponceau S staining.

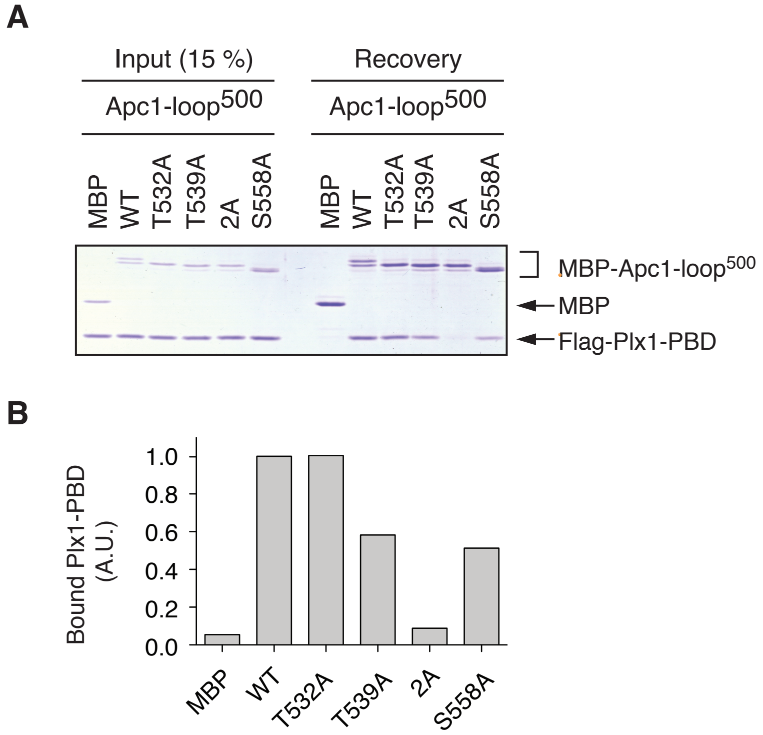

Appendix Fig S2. Binding of Plx1-PBD to mutant Apc1-loop500 proteins phosphorylated by Cdk2/cyclin A *in vitro*.
A. MBP-Apc1-loop500 WT or its derivatives (T532A, T539, 2A and S558A) (10 μg) was incubated in the presence of 1 mM ATP and Cdk2/cyclin A at 30˚C for 2 h. MBP-fused Apc1-loop500 was isolated by amylose beads and further incubated with WT Plx1-PBD (6 μg) on ice for 10 min. The bound proteins were recovered by amylose beads and analysed as described in (Fig 1D).
B. Quantification of bound Plx1-PBD in (A). The intensity of WT was arbitrarily set to 1.0.

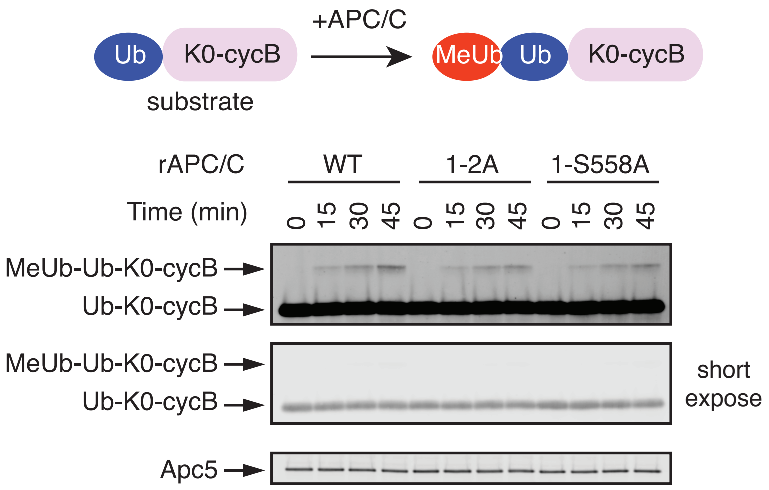

Appendix Fig S3. Ubiquitylation activity of mutant APC/Cs.
The recombinant WT APC/C or mutant APC/C (1- 2A or 1-S558A) was incubated with ΔAPC anaphase extract at 23˚C for 60 min. The recovered APC/C-Cdc20 complex was subjected to ubiquitylation assay using methylated ubiquitin and analysed by SDS–PAGE and immunoblotting with anti-Flag antibody. Flag-tagged ubiquitin-fused lysine-less cyclin B1-70 (referred to as Ub-K0-cyclin B) was used as a substrate. Quantification is presented in Fig 2C.

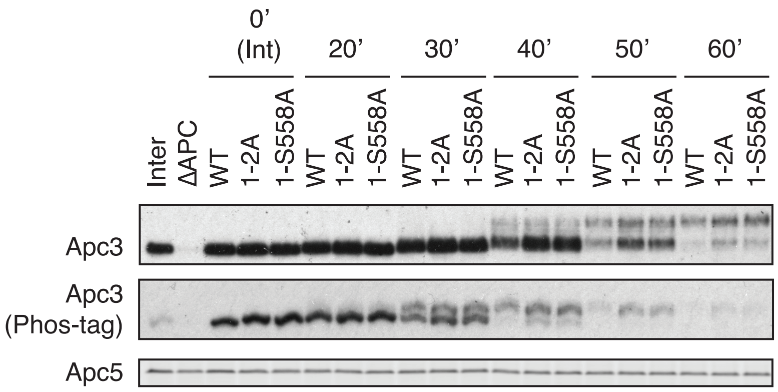

Appendix Fig S4. Delayed phosphorylation of Apc3 in mutant APC/Cs (1-2A and 1-S558A) during mitotic progression.
The purified recombinant WT APC/C or its derivatives (1- 2A and 1-S558A) was incubated with APC/C-depleted (ΔAPC) interphase extracts supplemented with non-degradable cyclin B at 23˚C. Samples taken at times shown after addition of cyclin B were analysed by SDS–PAGE and immunoblotting with indicated antibodies. The same set of samples was also analysed by SDS–PAGE in the presence of Phos-tag (middle).

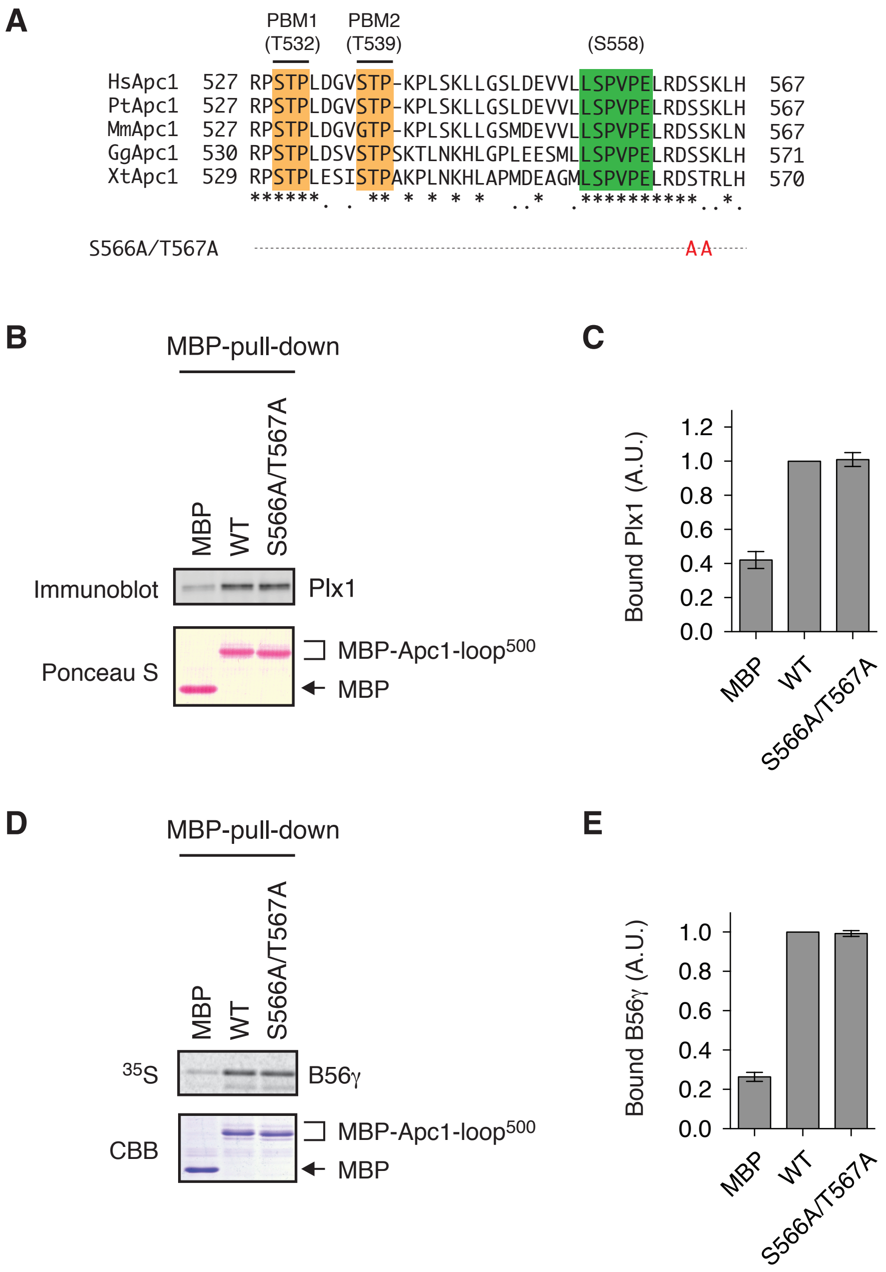

Appendix Fig S5. Phosphorylation of S566/T567 on Apc1-loop500 is dispensable for both Plx1- and B56γ-binding.
A. S566/T567 sites and their alanine mutations were shown in multiple alignment of sequences of Apc1-loop500 as Fig 1A.
B. Plx1-binding activity. MBP-fused Apc1-loop500 WT or its derivative (S566A/T567A) was incubated with anaphase extracts supplemented with non-degradable cyclin B at 23˚C for 1 h. The bound proteins were analysed as described in Fig 1B.
C. Quantification of (B). The bar graph is quantification of bound Plx1. The intensities of Apc1-loop500 WT were arbitrarily set to 1.0. Error bars, SEM from three independent experiments.
D. B56γ-binding activity. MBP-fused Apc1-loop500 WT or its derivative (S566A/T567A) was incubated with the ^{35}S-labelled Flag-B56γ in anaphase extracts supplemented with non-degradable cyclin B at 23˚C for 1 h. The bound proteins were analysed as described in Fig 3E.
E. Quantification of (D). The bar graph is quantification of bound B56γ. The intensities of WT were arbitrarily set to 1.0. Error bars, SEM from three independent experiments.

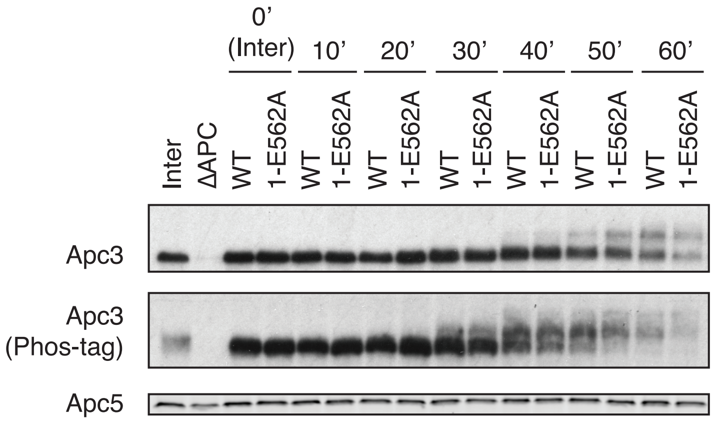

Appendix Fig S6. APC/C (1-E562A) mutation accelerates Apc3 phosphorylation in *Xenopus* mitotic egg extracts.

The purified recombinant WT APC/C or Apc1-loop500 mutant APC/C (1-E562A) was incubated with APC/C-depleted (ΔAPC) interphase extracts supplemented with non-degradable cyclin B at 23˚C. Samples taken at times shown after addition of cyclin B were analysed by SDS–PAGE and immunoblotting with indicated antibodies. The same set of samples was also analysed by SDS-PAGE in the presence of Phos-tag (middle).

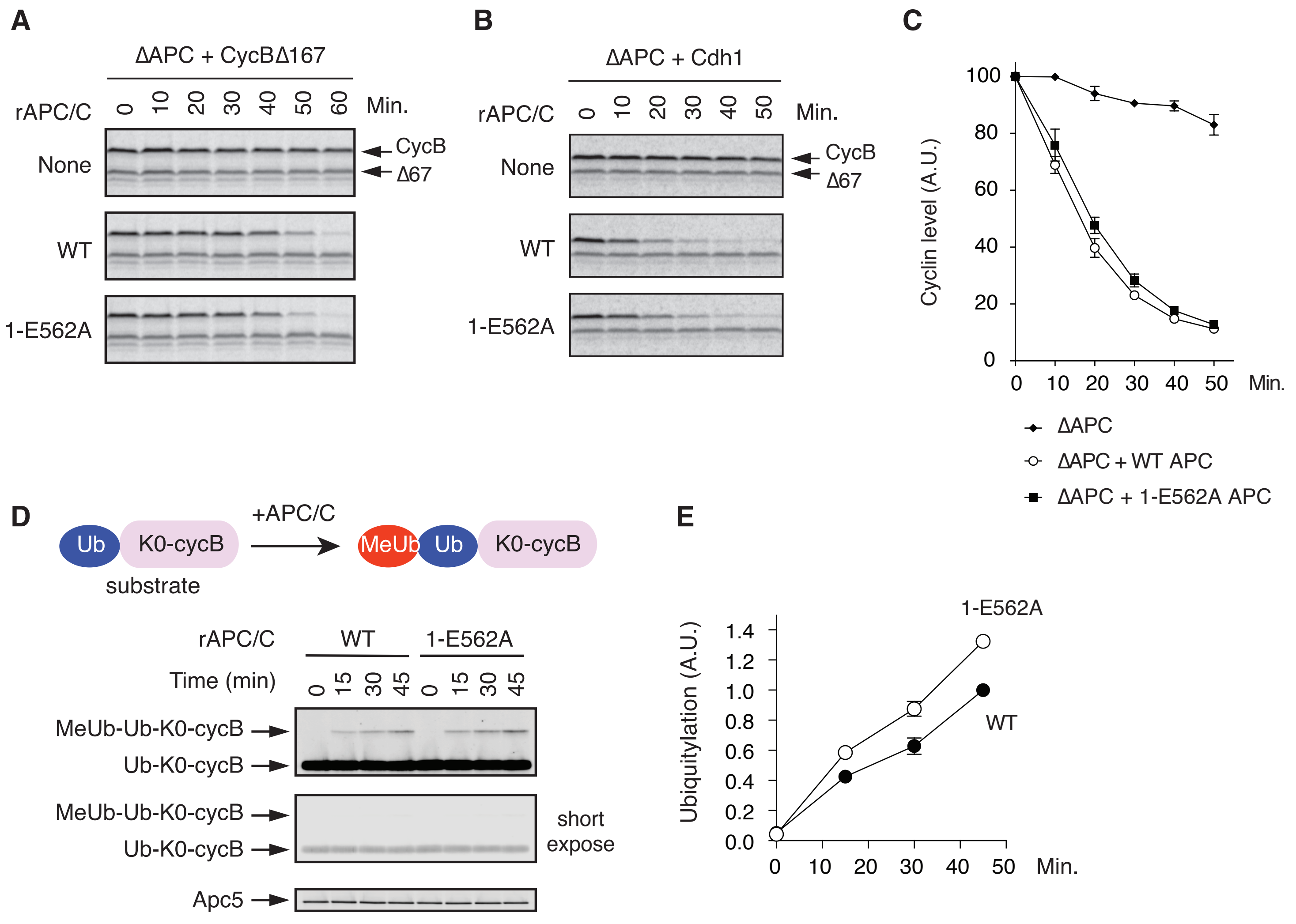

Appendix Fig S7. Apc1-loop500 mutant APC/C (1-E562A) is more active in anaphase than WT APC/C.
A. Cdc20-dependent destruction assay in anaphase. Mitotic activation of purified recombinant WT APC/C or Apc1 mutant APC/C (1-E562A) was examined using cyclin destruction assay. See Fig 6C.
B. Cdh1-dependent destruction assay. The purified recombinant WT APC/C or its mutant APC/C (1-E562A) was incubated with APC/C-depleted (ΔAPC) extracts supplemented with Cdh1 at 23˚C. ^{35}S-labelled cyclin B and a version of cyclin B lacking the N-terminal 67 residues (Δ67, stable control) were used as substrates. Samples taken at indicated time points after addition of substrates were analysed by SDS–PAGE and autoradiography.
C. Quantification of (B). Error bars, SEM from three independent experiments.
D. Ubiquitylation assay. The purified recombinant WT APC/C or its mutant APC/C (1-E562A) was incubated with Δ APC anaphase extract at 23˚C for 45 min. The recovered APC/C-Cdc20 complex was subjected to ubiquitylation assay as described in Fig 2C.
E. Quantification of (D). The intensity of WT at 45 min was arbitrarily set to 1.0. Error bars, SEM from three independent experiments.

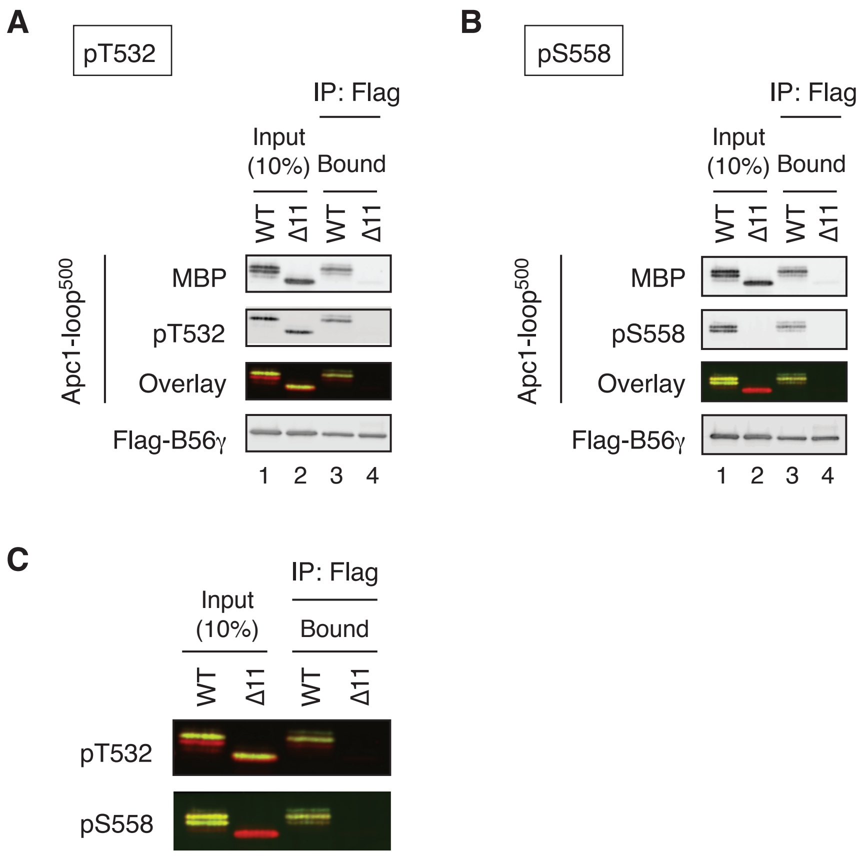

Appendix Fig S8. B56 subunit preferentially binds to the T532-phoshorylated Apc1-loop500.
A. Flag-tagged B56γ was incubated with the MBP-fused Apc1-loop500 WT or its deletion derivative (Δ11, deletion of 11 residues including the B56-binding motif) phosphorylated by Cdk2/cyclin A. The bound proteins to B56γ were recovered by anti-flag affinity M2 beads, separated by SDS–PAGE and detected by immunoblotting with phospho-specific (pT532), MBP or Flag antibody. The signals of a phospho-specific antibody (pT532) and MBP were pseudo-coloured (green; pT532, red; MBP) and overlaid (Overlay). No signal was detected from Δ11 Apc1-loop500 (lane 4) except the bait, Flag-tagged B56γ.
B. Same as A, but a phospho-specific antibody (pS558) was used.
C. Enlarged images of the overlay panel in A and B.
