# Supplementary material for: Dynamic regulation of mitotic ubiquitin ligase APC/C by coordinated Plx1 kinase and PP2A phosphatase action on a flexible Apc1 loop
Source: EMBO J. 2021 Jul 22;40(18):e107516. doi: 10.15252/embj.2020107516 (PMC8441438; doi:10.15252/embj.2020107516)
Supplement: Supplementary file 3 — Expanded View Figures PDF [file EMBJ-40-e107516-s003.pdf]

Expanded View Figures

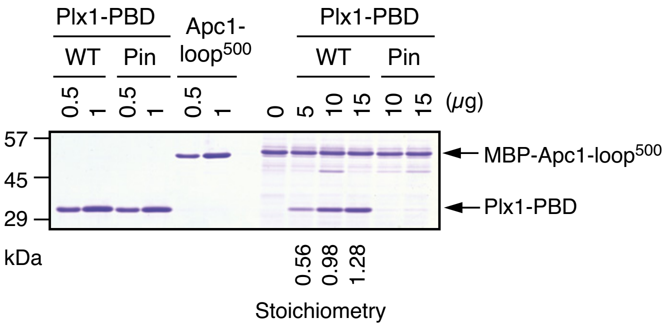

Figure EV1. Plx1-PBD, not its Pincer mutant, binds Apc1-loop^{500}.

Apc1-loop^{500} binding assay to Plx1-PBD. See Fig 1F for the details.

Figure EV2. Destruction assays using mutant APC/Cs (1–2A and 1-S558A).

A Cdc20-dependent destruction assay in anaphase. The purified recombinant WT APC/C or its derivatives (1–2A and 1-S558A) was incubated with its substrates (^{35}S-labelled cyclin B and a version of cyclin B lacking the N-terminal 67 residues, Δ67) in APC/C-depleted (ΔAPC) interphase extracts supplemented with non-degradable cyclin B at 23°C. Samples taken at indicated time points were analysed by SDS–PAGE and autoradiography.

B Quantification of (A). The relative cyclin levels are shown, normalised with reference to the intensities found at time 0 for each time point. Error bars, SEM from three independent experiments.

C Cdh1-dependent destruction assay. The purified recombinant WT APC/C or its derivatives (1–2A and 1-S558A) was incubated with its substrates in APC/C-depleted (ΔAPC) interphase extracts supplemented with Cdh1 at 23°C. Samples taken at indicated time points were analysed by SDS–PAGE and autoradiography.

D Quantification of (C). The relative cyclin levels are shown, normalised with reference to the intensities found at time 0 for each time point. Error bars, SEM from three independent experiments.

© 2021 The Authors

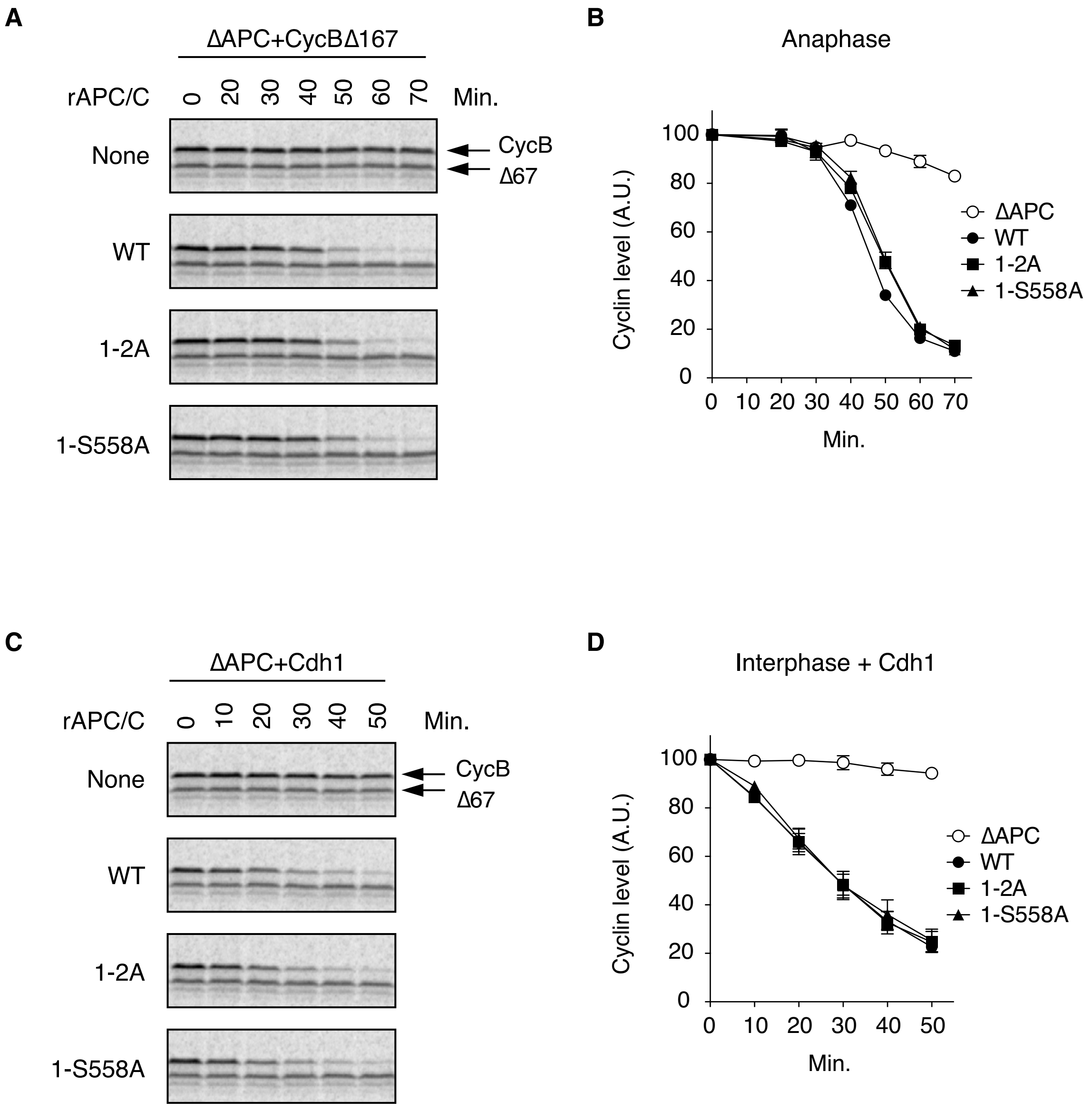
A
ΔAPC+CycBΔ167
rAPC/C
0
20
30
40
50
60
70
Min.
None
WT
1-2A
1-S558A
CycB
Δ67
B
Anaphase
Cyclin level (A.U.)
100
80
60
40
20
0
0
10
20
30
40
50
60
70
Min.
ΔAPC
WT
1-2A
1-S558A
C
ΔAPC+Cdh1
rAPC/C
0
10
20
30
40
50
Min.
None
WT
1-2A
1-S558A
CycB
Δ67
D
Interphase + Cdh1
Cyclin level (A.U.)
100
80
60
40
20
0
0
10
20
30
40
50
Min.
ΔAPC
WT
1-2A
1-S558A

Figure EV2.

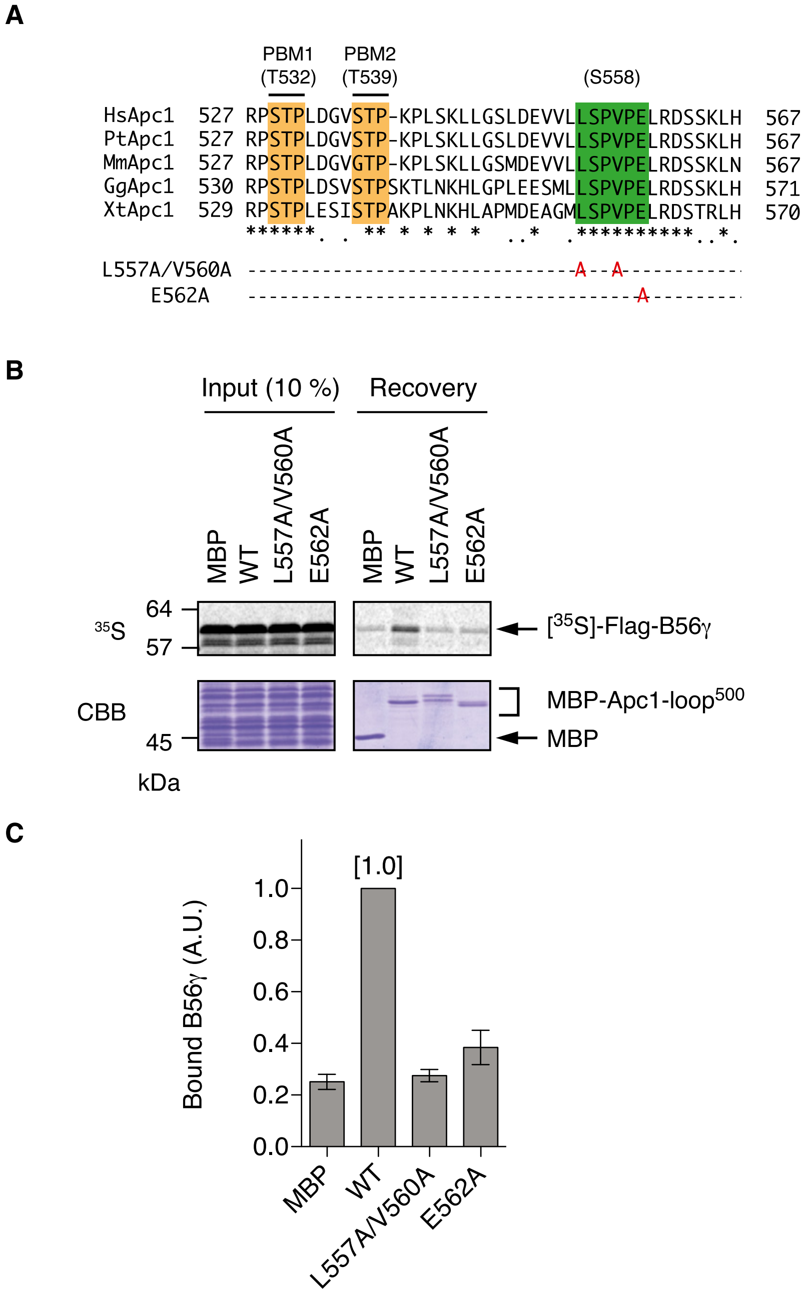

Figure EV3. Mutations in the B56-binding motif abolish B56 and Apc1-loop^{500} interaction.

A B56-binding site mutations (L557A/V560A and E562A) were shown in multiple alignment of sequences of Apc1-loop^{500} as Fig 1A.

B Binding assay using MBP-fused Apc1-loop^{500} fragments and B56γ. MBP-fused Apc1-loop^{500} WT or its derivatives (L557A/V560A or E562A) was incubated with the ^{35}S-labelled Flag-B56γ in anaphase extracts supplemented with non-degradable cyclin B at 23°C for 1 h. The bound proteins were recovered by amylose beads, separated by SDS–PAGE and detected by autoradiography or Coomassie brilliant blue (CBB) staining.

C Quantification of (B). The bar graph is quantification of bound B56γ. The intensities of WT were arbitrarily set to 1.0. Error bars, SEM from three independent experiments.

© 2021 The Authors

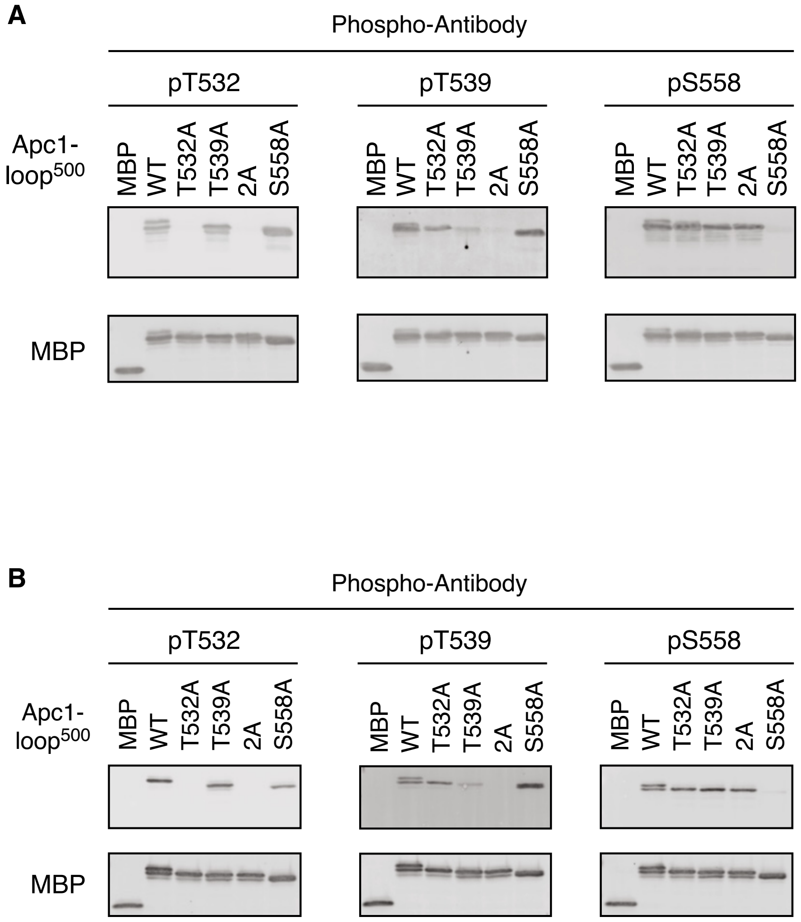

Figure EV4. Specificities of phospho-specific antibodies.

A MBP-fused Apc1-loop^{500} WT or its derivatives (T532A, T539A, 2A and S558A) was incubated with anaphase extracts supplemented with non-degradable cyclin B at 23°C for 1 h. The bound proteins were recovered by amylose beads, separated by SDS–PAGE and detected by immunoblotting with a phospho-specific antibody (pT532, pT539 or pS558) and MBP antibody.

B Phospho-specific antibodies recognise CDK sites of Apc1-loop^{500} phosphorylated by Cdk2/cyclin A *in vitro*. MBP-fused Apc1-loop^{500} WT and its derivatives (T532A, T539A, 2A and S558A) recovered in Appendix Fig S2 were analysed as described in A.

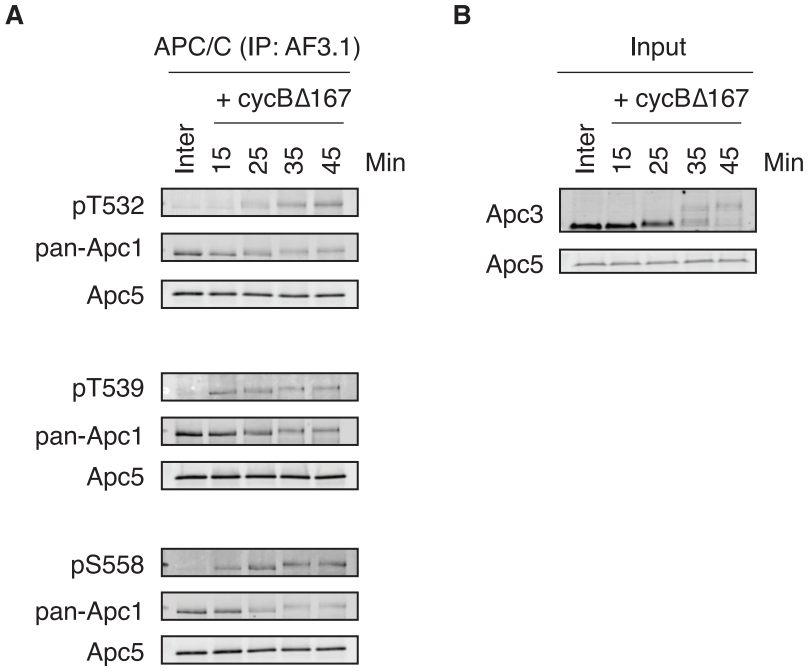

Figure EV5. Phosphorylation kinetics of T532, T539 and S558 on Apc1-loop^{500} of the endogenous APC/C in *Xenopus* egg extracts.

A Endogenous APC/C was immunoprecipitated with Apc3 monoclonal antibody (AF3.1) beads after incubation at 23°C for the indicated time points after mitotic induction by adding non-degradable cyclin BΔ167. The bound proteins were analysed by SDS–PAGE and immunoblotting with indicated antibodies. See Fig 5C for quantification. "Inter" denotes interphase.

B Monitoring of mitotic induction from interphase extract upon cyclin B addition. When Cdk1 becomes active, Apc3 is hyper-phosphorylated, as observed with the band shifts of Apc3. Samples were taken at the indicated times and analysed by SDS–PAGE and immunoblotting with indicated antibodies.

© 2021 The Authors
